# A Novel Molecular Profile of Hormone‐Sensitive Prostate Cancer Defines High Risk Patients

**DOI:** 10.1002/cam4.70472

**Published:** 2025-02-20

**Authors:** Claudia Piombino, Cecilia Nasso, Stefania Bettelli, Samantha Manfredini, Maria Giuseppa Vitale, Stefania Pipitone, Cinzia Baldessari, Matteo Costantini, Albino Eccher, Ilenia Mastrolia, Virginia Catani, Francesca Bacchelli, Stefania Ferretti, Massimo Dominici, Roberto Sabbatini

**Affiliations:** ^1^ Division of Oncology, Department of Oncology and Hematology University Hospital of Modena Modena Italy; ^2^ Division of Oncology S. Corona Hospital Pietra Ligure Italy; ^3^ Division of Molecular Pathology and Predictive Medicine University Hospital of Modena Modena Italy; ^4^ Department of Pathology University Hospital of Modena Modena Italy; ^5^ Laboratory of Cellular Therapy, Division of Oncology, Department of Medical and Surgical Sciences for Children & Adults University of Modena and Reggio Emilia Modena Italy; ^6^ Clinical Trials Office, Division of Oncology, Department of Medical and Surgical Sciences for Children & Adults University of Modena and Reggio Emilia Modena Italy; ^7^ Urology Unit University Hospital of Modena Modena Italy

**Keywords:** *AKT2*, *FOS*, metastatic hormone‐sensitive prostate cancer, NanoString nCounter PanCancer Pathways Panel, *NR4A1*

## Abstract

**Background:**

The therapeutic management of metastatic hormone‐sensitive prostate cancer (mHSPC) is still based on clinical and pathological parameters due to the lack of biomarkers that may drive tailored treatment.

**Methods:**

In this non‐randomized, single‐center, retrospective trial, we searched for a genetic signature using the NanoString nCounter PanCancer Pathways Panel on formalin‐fixed paraffin embedded prostate cancer samples belonging to 48 patients with *de novo* or relapsed mHSPC. Patients were divided into a high‐clinical‐risk group (*n* = 36) and a low‐clinical‐risk group (*n*&amp;#x02009;=&amp;#x02009;12) according to the mean time to metastatic relapse.

**Results:**

The analysis of Nanostring nCounter Panel data revealed differential expression of 42 genes between high‐clinical‐risk and low‐clinical‐risk groups. All the genes except for *NR4A1* and *FOS* were upregulated in the high‐clinical‐risk group. A general overexpression of apoptosis, PI3K and MAPK pathway‐related genes, including AKT2, was observed in the high‐clinical‐risk group.

**Conclusion:**

The differential genetic signature identified between the two study groups revealed novel biomarkers in mHSPC, additionally suggesting new therapeutic targets within the hormone sensitive phase, such as *AKT2*. Further prospective larger cohort studies are needed to assess the prognostic value of our findings and their exact role in prostate cancer progression.

**Abbreviations**
adj: adjusted
ADT: androgen deprivation therapy
AR: androgen receptor
FFPE: formalin‐fixed paraffin‐embedded
IEGs: immediate early genes
IQR: interquartile range
mCRPC: metastatic castration‐resistant prostate cancer
mHSPC: metastatic hormone‐sensitive prostate cancer
mOS: median overall survival
mPFS1: median PFS to first line treatment
NHAs: new hormonal agents
OS: overall survival
PC: prostate cancer
PFS: progression‐free survival
PSA: prostate specific antigen
SD: standard deviation

## 1 Introduction

Hormone sensitivity is a key feature of most prostate cancer (PC). The binding of testosterone and 5α‐dihydrotestosterone to the androgen receptor (AR) induces tumor growth and progression by AR homodimerization and interaction with accessory proteins and consequent AR binding to the promoter regions of genes involved in cell proliferation and apoptosis evasion. Simultaneously, a rapid non‐genomic signaling pathway, initiated by the association of AR with molecular substrates outside the nucleus, contributes to cell proliferation by activation of MAPR/ERK and PI3K/AKT pathways and by exclusion of other steroid receptors from the nucleus. Therefore, the decrease in serum androgens achieved by castration or androgen deprivation therapy (ADT) induces tumor regression by targeting AR [1, 2, 3, 4].

It has been estimated that up to one third of patients with localized PC undergoing prostatectomy or radiation therapy will develop metastatic recurrence after local treatment [5]. In addition, approximately 5%–10% of patients are diagnosed with *de novo* metastatic PC. Although these different clinical presentations, all those patients are supposed to be responsive to ADT, a condition that is known as metastatic hormone‐naïve or hormone‐sensitive PC (mHSPC). During ADT, patients affected by mHSPC generally show a variable prostate specific antigen (PSA) decrease, often associated with the clinical and radiological response; however, some patients rapidly progress to a castration‐resistant phase with subsequent poor prognosis [4].

Since 2015, the prognosis of mHSPC has slightly improved thanks to the introduction of new hormonal agents (NHAs) and chemotherapy combined with ADT from the first‐line setting [6, 7, 8, 9, 10]. More recently, the phase III trials PEACE‐1 and ARASENS have investigated the role of triplet therapy in mHSPC combining docetaxel, abiraterone, and ADT, and docetaxel, darolutamide, and ADT, respectively. Both trials showed an increase in overall survival (OS) compared with standard combination therapies [11, 12]. In this complex and evolving landscape, where clinicians have multiple effective therapeutic options for treating mHSPC, the priority is to determine which treatment combination and sequence is best suited to each patient, considering comorbidities and preferences. However, the therapeutic management in this context is still based only on clinical and pathological aspects, and no head‐to‐head trial of NHAs and docetaxel has been planned to our knowledge.

Although few biomarkers have been identified in mHSPC, several genetic alterations discovered in metastatic castration‐resistant prostate cancer (mCRPC), associated with aggressiveness or metastatic spread, are also detectable in mHSPC at a similar prevalence. For example, the incidence of aberrations in *PTEN*, *TP53*, *FOXA1*, *PI3K*, *APC*, and *BRCA2* does not differ significantly between *de novo* mHSPC and mCRPC [13, 14]. Some of these alterations have been associated with response to specific treatments in mCRPC [15]. The availability of prognostic and predictive biomarkers that could help clinicians in the choice of the most appropriate treatment for each patient affected by mHSPC is still an unmet clinical need.

Several methods have historically been applied for the molecular characterization of PC patients. A rich array of diagnostic and prognostic tests has emerged for serum (4K, phi), urine (Progensa, T2‐ERG, ExoDx, SelectMDx), and tumor tissue (ConfirmMDx, Prolaris, Oncoytype DX, Decipher) [16]. NanoString nCounter is a multiplex nucleic acid hybridization technology that enables a reliable and reproducible assessment of the expression of up to 800 genes in 12 samples in a single assay. It does not require the conversion of mRNA to cDNA or the amplification of the resulting cDNA, thus overcoming some common constraints associated with the use of the polymerase chain reaction [17]. Different from RNA sequencing, NanoString nCounter does not require high‐quality RNA, and so it can be used with the formalin‐fixed paraffin‐embedded (FFPE) material [18].

Our study wants to evaluate the presence of a prognostic genetic signature using the nCounter PanCancer Pathways Panel (NanoString panel) in a cohort of patients with *de novo* or metachronous mHSPC, with the additional aim of giving novel insights into the molecular characterization of mHSPC.

## 2 Materials and Methods

### 2.1 Patient Cohort

A total of 48 patients with *de novo* or metachronous mHSPC were recruited. For each patient, a FFPE archival PC specimen was selected, including both primary PC and metastatic samples. Tumor specimens were acquired between 2007 and 2020 and were retrospectively identified using institutional pathology and clinical databases from the University Hospital of Modena. Retrospective review of medical records including clinical and pathological characteristics (median age at diagnosis of localized and metastatic disease, stage at first diagnosis, grading according to ISUP Grade Group, PSA level at diagnosis of mHSPC, sites of metastasis) was performed. The study population was subsequently divided into a high‐clinical‐risk group (*N* = 36) and a low‐clinical‐risk group (*N* = 12) according to the mean time to metastatic relapse. Institutional ethical approval by the Area Vasta Emilia Nord Ethics Committee was granted for the analysis of archival tissue and clinical data according to the Declaration of Helsinki principles (approval number 927/2020, date of approval November 3, 2020). Informed consent was obtained by patients recruited.

### 2.2 The NanoString Assay

Tumor‐representative areas containing at least 50% tumor cells were isolated by manual micro‐dissection. RNA was extracted from 10 μm‐thick sections of FFPE tissues using the MagCore Total RNA FFPE One‐Step Kit by using the MagCore instrument (all by RBC Bioscience). The nCounter PanCancer Pathways Panel assesses expression of 770 genes involved in the major cancer‐related pathways (Wnt, Hedgehog, apoptosis, cell cycle, RAS, PI3K, STAT, MAPK, Notch, TGF‐β, chromatin modification, transcriptional regulation, and DNA damage control). NanoString technology is based on direct molecular bar‐coding and digital detection of individual target molecules using a unique probe pair for each target of interest. The probe pair consists of a color‐coded Reporter probe, which carries the visible signal on its 5′‐end, and a Capture probe, which carries a covalently attached biotin moiety on the 3′‐end. Each Reporter probe has six positions, and each position can be one of four colors. A large diversity of probes can be mixed with the sample in a single well, with each Reporter probe interacting with a specific target. These target–probe complexes are then individually resolved and identified during data collection (Figure S1). Probes were placed into a reaction in massive excess to target nucleic acid molecules to ensure that each target finds a probe pair. After overnight hybridization, excess probes were washed away using a two‐step magnetic bead‐based purification on the nCounter PrepStation instrument. These magnetic beads are derivatized with short nucleic acid sequences that are complementary to the Capture probes or the Reporter probes and are used sequentially. First, the hybridization mixture was allowed to bind to the magnetic beads complementary to the Capture probes. Wash steps were performed to remove excess Reporter probes and non‐target cellular transcripts. The target–probe complexes and unbound Capture probes were then eluted off the beads and allowed to bind to a second set of beads complementary to the Reporter probes. Wash steps were performed to remove excess Capture probes. Finally, the purified target–probe complexes were eluted off the beads and were immobilized and aligned for data collection.

Data collection was performed on an nCounter Digital Analyzer using epifluorescence microscopy and CCD capture technology to yield hundreds of thousands of target molecule counts. Digital images are processed within the instrument, and the Reporter probe raw counts were tabulated for data analysis with NanoString's nSolverTM Analysis Software.

### 2.3 Statistical Analyses

Clinical characteristics were reported as median (interquartile range, IQR) or average (standard deviation, SD) for continuous variables and as absolute frequency (%) for categorical variables. High‐clinical‐risk and low‐clinical‐risk groups were compared using the *t*‐test or Mann–Whitney test for the continuous variables and Pearson Chi square or Fisher's exact test for the categorical variables. Concerning time‐to‐event variables (progression‐free survival, PFS, and OS), results were reported as median (95% confidence interval; 95% CI), and Kaplan–Meier curves were presented; to test the equality of survivor functions, the log‐rank test was used. All statistical analyses were performed with statistical software Stata version 16.0 (Stata Corporation, College Station, TX, USA), and *p*‐values < 0.05 were considered statistically significant. The differential gene expression between high‐ and low‐clinical‐risk groups was reported as log2‐fold change; while a positive fold change value indicates an increase in expression, a negative fold change indicates a decrease in expression. The false discovery rate was controlled by Benjamini–Yekutieli procedure, and adjusted (adj) *p*‐values < 0.05 were considered statistically significant.

## 3 Results

### 3.1 High‐Clinical‐Risk and Low‐Clinical‐Risk Groups Have Distinct Clinical Outcomes

A total of 48 patients diagnosed with mHSPC from 2007 to 2020 at Modena Cancer Centre were enrolled. The study population was divided into a high‐clinical‐risk group (*N* = 36) and a low‐clinical‐risk group (*N* = 12) according to the mean time to metastatic relapse, which was respectively 3 and 135 months (*p* < 0.001). The high‐clinical‐risk group included almost entirely *de novo* mHSPC (33/36, 92%) with only three patients experiencing metastatic relapse within 3 years of the first diagnosis. In contrast, patients belonging to the low‐clinical‐risk group experienced metastatic relapse more than 6 years after radical prostatectomy (range 72–198 months).

The analysis of the study population according to clinicopathological characteristics is reported in Table 1. The median age at first PC diagnosis was not statistically different between the two groups, but patients belonging to the high‐clinical‐risk group were significantly younger at the diagnosis of mHSPC than those belonging to the second group (68 vs. 77 years, *p* < 0.001). The two groups were homogeneous for ISUP Grade Group and Eastern Cooperative Oncology Group performance status. Analyzing the burden of disease at metastatic diagnosis according to CHAARTED criteria, 86% of patients in the high‐clinical‐risk group and 33% in the low‐clinical‐risk group were classified as high‐volume (*p* < 0.001). Moreover, the PSA level at the diagnosis of mHSPC was sensibly lower in the low‐clinical‐risk group compared to the high‐clinical‐risk group (*p* < 0.001).

**TABLE 1:** Baseline patient characteristics.

	High‐risk group (*N* = 36)	Low‐risk group (*N* = 12)	*p*
Mean time to metastatic relapse (months, SD)	3 (10)	135 (40)	< 0.001
Median age at first diagnosis (years, IQR)	68.4 (62.7–73.3)	64.6 (63.0–69.7)	0.41
Median age at diagnosis of mHSPC (years, IQR)	68.4 (62.8–73.4)	77.1 (73.6–81.2)	< 0.001
PS ECOG (*N*, %)			0.13
0–1	33 (92)	9 (75)	
2	3 (8)	3 (25)	
Stage at first diagnosis (*N*, %)			< 0.001
II–III	3 (8)	12 (100)	
IV	33 (92)	0	
ISUP grade group (*N*, %)			0.42
≤ 3	6 (18)	0	
4	14 (41)	4 (33)	
5	14 (41)	8 (67)	
NA	2	0	
PSA at diagnosis of mHSPC (*N*, %)			< 0.001
≤ 20 ng/mL	10 (28)	11 (92)	
> 20 ng/mL	26 (72)	1 (8)	
High‐volume disease (*N*, %)	31 (86)	4 (33)	< 0.001
Mean time to castration resistance (months, SD)	12.7 (10)	51 (44)	< 0.001

**FIGURE 1:**
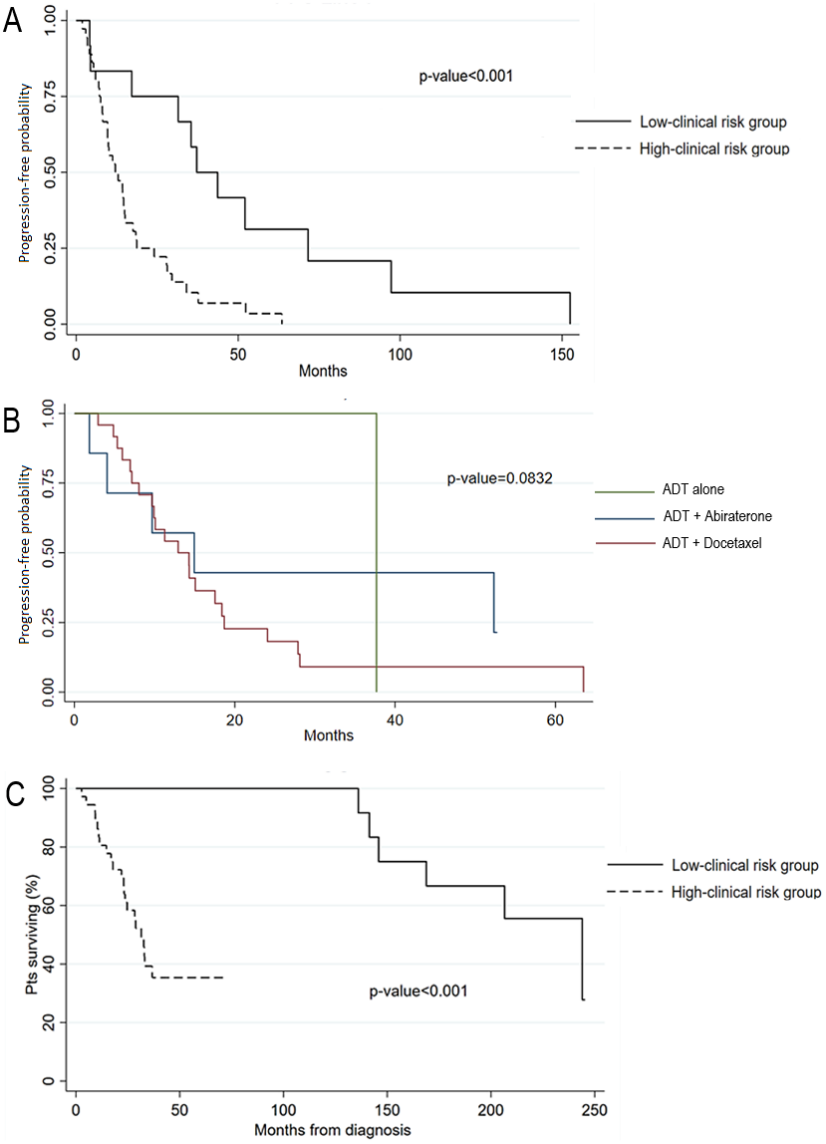
Survival analysis: (A) PFS1 in the low‐clinical‐risk group vs. the high‐clinical‐risk group, (B) PFS1 according to first‐line therapy in the high‐clinical‐risk group, and (C) OS in the low‐clinical‐risk group vs. the high‐clinical‐risk group.

After a median follow‐up of 35 months, only two patients (one for each group) were still receiving a first‐line treatment. The median PFS to first line treatment (mPFS1) was 12.1 months (95% CI: 8.2–15.1) in the high‐clinical‐risk group versus 37.2 months (95% CI: 4.4–71.6) in the low‐clinical‐risk group (*p* < 0.001) (Figure 1A). No statistically significant difference in terms of mPFS1 was found in the high‐clinical‐risk group according to the first‐line therapy received (ADT alone or in association with docetaxel or abiraterone) (Figure 1B). The mean time to castration resistance, considered as the average time from the start of ADT to radiological, biochemical, or clinical progression (with testosterone levels ≤ 0.5 nmol/L) was 14 months in the high‐clinical‐risk group versus 49.5 months in the low‐clinical‐risk group (*p* = 0.005). During cutoff survival analysis, 28 patients died. The mOS (considered as time from the diagnosis of mHSPC to death for any cause) was 31 months (95% CI: 23; not reached) in the high‐risk group versus 244 months (95% CI: 141; not reached) in the low‐risk group (*p* < 0.001) (Figure 1C).

### 3.2 NanoString Panel Identifies a New Prognostic Genetic Signature

**FIGURE 2:**
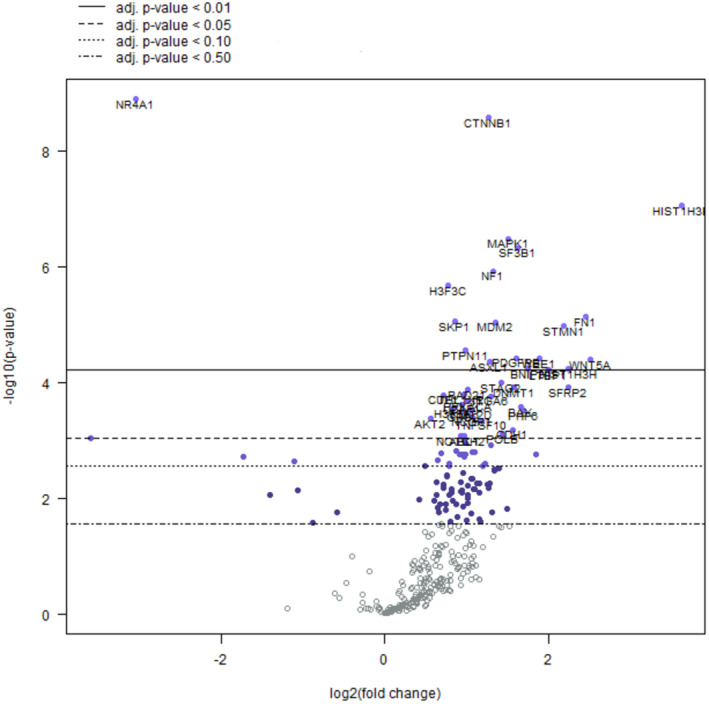
Volcano plot of the PanCancer Pathway gene panel. Volcano plot allows to evaluate for each target gene (represented by a dot) the different expression in the two groups (log2‐fold change, on the *x*‐axis) and its level of significance (−log10 *p*‐value, on the *y*‐axis). The horizontal lines represent the thresholds of adjusted (adj) *p*‐values according to Benjamini–Yekutieli. The overexpressed targets in the high‐clinical‐risk group compared to the low‐clinical‐risk group are on the right side (positive log2‐fold change), while the downregulated targets are on the left side (negative log2‐fold change). The more the target is in the upper part, the greater the statistical significance. The color of the dots reflects the level of statistical significance: white dots represent genes not significantly differentially expressed between the two groups, while colored dots represent genes significantly differentially expressed between the two groups (adj *p*‐value < 0.05). Among colored dots, the lighter the color, the greater the statistical significance.

**FIGURE 3:**
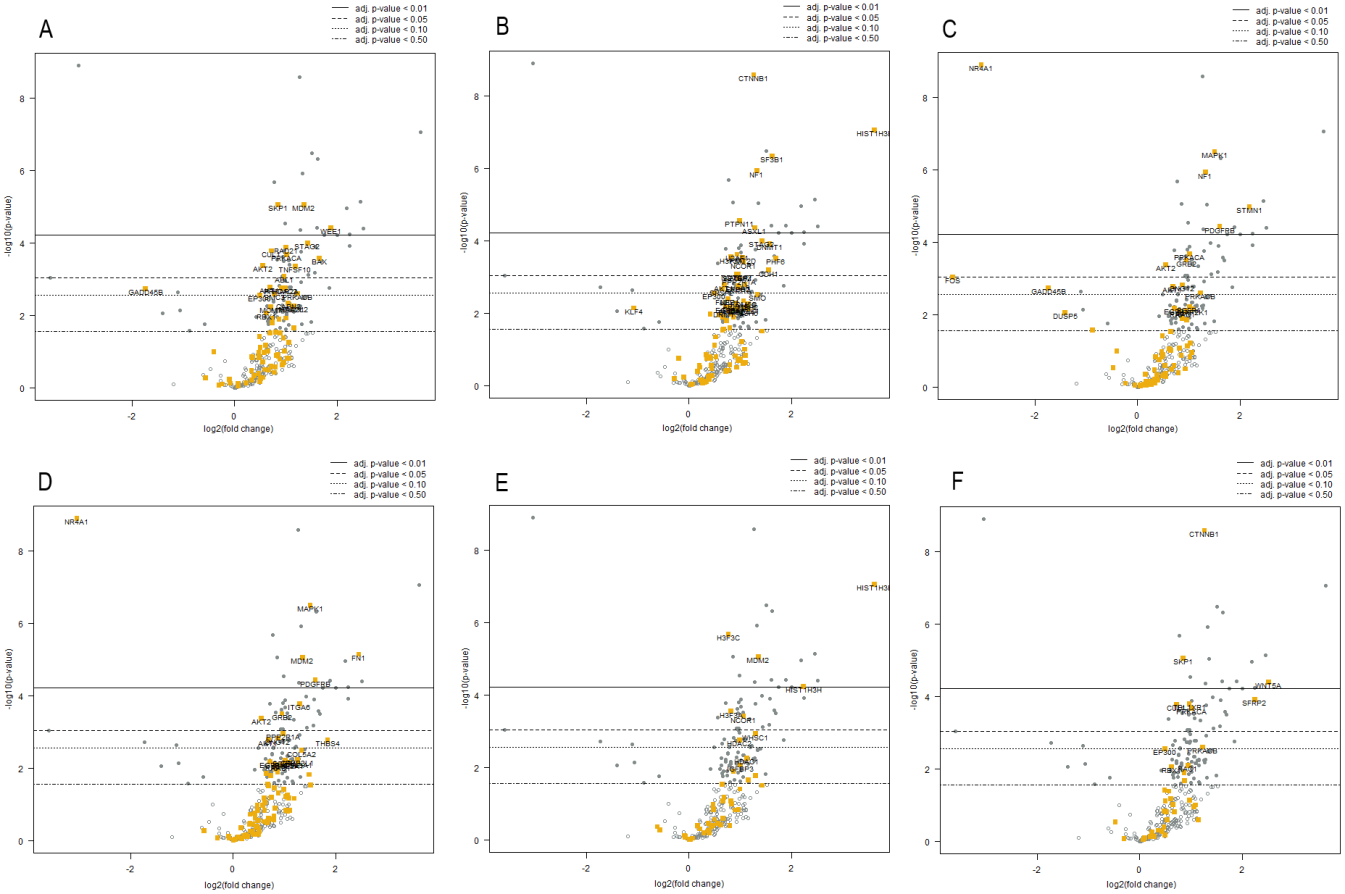
Volcano plots for selected gene sets. The genes included in the set are represented by yellow squares, while dots represent other genes included in the PanCancer Pathway gene panel (white dots: genes not significantly differentially expressed between the two groups; gray dots: genes significantly differentially expressed between the two groups). (A) Apoptosis gene set. The only downregulated gene in the high‐clinical‐risk group is GADD45B, without reaching the statistical significance. All targets with significant differences in expression are upregulated in the high‐risk clinical group: SKP1 (*p* = 8.86 × 10^−6^), MDM2 (*p* = 9.04 × 10^−6^), WEE1 (*p* = 3.89 × 10^−5^), STAG2 (*p* = 1.03 × 10^−4^), RAD21 (*p* = 1.32 × 10^−4^), CUL1 (*p* = 1.66 × 10^−4^), PRKACA (*p* = 2.13 × 10^−4^), BAX (*p* = 2.66 × 10^−4^), AKT2 (*p* = 4.23 × 10^−4^), and TNFSF10 (*p* = 4.53 × 10^−4^). (B) Driver gene set. The only downregulated gene in the high‐clinical‐risk group is KLF4, which does not reach the statistical significance. All targets with significant differences in expression are upregulated in the high‐clinical‐risk group: CTNNB1 (*p* = 2.67 × 10^−9^), HIST1H3B (*p* = 8.85 × 10^−8^), SF3B1 (*p* = 4.77 × 10^−7^), and NF1 (*p* = 1.20 × 10^−6^). (C) MAPK gene set. NR4A1 (*p* = 1.29 × 10^−9^) and FOS (*p* = 9.24 × 10^−4^) are downregulated in the high‐clinical‐risk group; MAPK1 (*p* = 3.30 × 10^−7^), NF1 (*p* = 1.20 × 10^−6^), STMN1 (*p* = 1.08 × 10^−5^), PDGFRB (*p* = 3.79 × 10^−5^), PRKACA (*p* = 2.13 × 10^−4^), GRB2 (*p* = 3.17 × 10^−4^), and AKT2 (*p* = 4.23 × 10^−4^) are upregulated in the high‐clinical‐risk group. (D) PI3K gene set. MDM2 is overexpressed (*p* = 9.04 × 10^−6^) and NR4A1 is downregulated (*p* = 1.29 × 10^−9^) in the high‐clinical‐risk group. (E) Transcriptional mis‐regulation gene set. All targets of the gene set are overexpressed in the high‐clinical‐risk group: HIST1H3B (*p* = 8.85 × 10^−8^), H3F3C (*p* = 2.13 × 10^−6^), MDM2 (*p* = 9.04 × 10^−6^), HIST1H3H (*p* = 5.90 × 10^−5^), H3F3A (*p* = 2.88 × 10^−4^), and NCOR1 (*p* = 3.96 × 10^−4^). (F) WNT gene set. *CTNNB1* (*p =* 2.67 × 10^−9^), *SKP1* (*p =* 8.86 × 10^−6^), *WNT5A* (*p =* 4.05 × 10^−5^), *SFRP2* (*p =* 1.22 × 10^−4^), *TBL1XR1* (*p =* 1.62 × 10^−4^), and *CUL1* (*p =* 1.66 × 10^−4^) are upregulated in the high‐clinical‐risk group.

Among 770 genes examined with the NanoString nCounter Gene Expression assay, a total of 42 genes were differentially expressed between the two groups (adj p‐value according to Benjamini–Yekutieli < 0.05); all these genes except two (*NR4A1* and *FOS*) were upregulated in the high‐clinical‐risk group (Figure 2, Table S1). A further analysis for specific gene sets identified according to specific pathways, analogy of function, or involvement in specific cellular activities was carried out using NanoString nSolver software. A general overexpression, especially in apoptosis, PI3K and MAPK pathway‐associated gene sets, was observed in the high‐clinical‐risk group compared to the low‐clinical‐risk group (Figure 3). More specifically, in the apoptosis gene set, the only downregulated gene was *GADD45B*, without reaching a statistical significance. All targets with significant differences in expression were upregulated with increasing order of adj *p*‐values: *SKP1*, *MDM2*, *WEE1*, *STAG2*, *RAD21*, *CUL1*, *PRKACA*, *BAX*, *AKT2*, and *TNFSF10* (Figure 3A). In the driver gene set, i.e., genes responsible for cancer development and progression, the only downregulated gene was *KLF4*, which however does not reach statistical significance, and all target genes with statistically significant differences were upregulated. In detail, *CTNNB1*, *HIST1H3B*, *SF3B1*, and *NF1* reached the most significant difference values (Figure 3B). In the MAPK pathway‐associated gene set, *NR4A1* and *FOS* were downregulated, while *MAPK1*, *NF1*, *STMN1*, *PDGFRB*, *PRKACA*, *GRB2*, and *AKT2* were upregulated in the high‐risk group (Figure 3C). The PI3K pathway‐associated gene set largely mirrored those observed for the MAPK gene set. Interestingly, this gene set revealed overexpression of *MDM2* and downregulation of *NR4A1* (Figure 3D). The transcriptional mis‐regulation gene set was also considered, revealing an increase in expression in the high‐clinical‐risk group of *HIST1H3B*, *H3F3C*, *MDM2*, *HIST1H3H*, *H3F3A*, and *NCOR1* (Figure 3E). The WNT gene set was finally assessed, revealing an upregulation of *CTNNB1*, *SKP1*, *WNT5A*, *SFRP2*, *TBL1XR1* and *CUL1* in the high‐risk group (Figure 3F).

## 4 Discussion

Determining the best treatment combination and sequence for each PC patient should be a priority for the clinicians. In the era of “precision medicine”, it is increasingly evident that clinical and pathological criteria are not enough to support which therapy is best suited for each patient affected by mHSPC. With the aim to identify a potentially prognostic genetic signature, we retrospectively selected 48 patients with *de novo* or relapsed mHSPC, further subdivided in high‐ and low‐clinical‐risk groups according to the mean time to metastatic relapse. To shed new light on prognosis and molecular characteristics, we used the NanoString nCounter assay to assess the expression of 770 genes in 48 archival PC specimens belonging to the two risk groups (36 from the high‐risk group and 12 from the low‐risk group). Interestingly, a general genes' expression upregulation was observed in the high‐clinical‐risk group compared to the low‐clinical‐risk group, except for two downregulated genes, namely, *NR4A1* and *FOS*.

NR4A1 (also known as Nur77 or NGFIB) is an orphan nuclear receptor that acts as a ligand‐independent transcription factor [19]. Studying tumorigenesis by patient‐derived breast cancer cells, Guo et al. identify NR4A1 as a master regulator of replication stress‐induced immediate early genes (IEGs) expression [20]. The physiological rapid induction of IEGs in response to replication stress guarantees coordinated proliferative and damage responses [21]. In normal conditions, NR4A1 localizes across IEGs gene bodies, inhibiting IEG transcription. Upon acute replication stress, NR4A1 dissociates from gene bodies, leading to IEG expression. *NR4A1* overexpression enhances breast tumorigenesis, while its deletion leads to massive chromosomal instability and proliferative failure, driven by increased expression of its IEG target FOS. In detail, protein kinase A (PKA), ERK, p38, MAPK, Rho‐A‐actin, PI3K, and other kinases phosphorylate multi‐protein complexes (SRF, CREB) that bound the IEG promoters, encoding their transcription factors (FOS, FOSB, EGFR1, NR4A1). Interestingly, NR4A1 has also been a regulator of T cell exhaustion, mechanism implied in the response to immunotherapy [20].

TCGA and other public clinical databases reveal distinct correlations between *NR4A1* expression and NR4A1‐IEG chromatin accessibility. Over half of all primary human cancers, including virtually all PC and the more favorable subsets of breast cancer, have retained open chromatin domains at IEG gene bodies, denoting a favorable clinical prognosis [22]. The NR4A1‐IEG axis may be therefore lost in aggressive cancer. In our study, high‐clinical‐risk mHSPC showed *NR4A1* and *FOS* downregulation; however, we did not have information regarding open chromatin domains at IEG gene bodies. The loss of open chromatin domains at IEG gene bodies could impair the NR4A1‐IEG axis irrespective of *NR4A1* expression and consequently lead *FOS* downregulation. Nevertheless, NR4A1 regulates a wide range of processes including metabolism, angiogenesis, inflammation, and immune cell differentiation, which could be involved in tumor initiation and progression [19].

**FIGURE 4:**
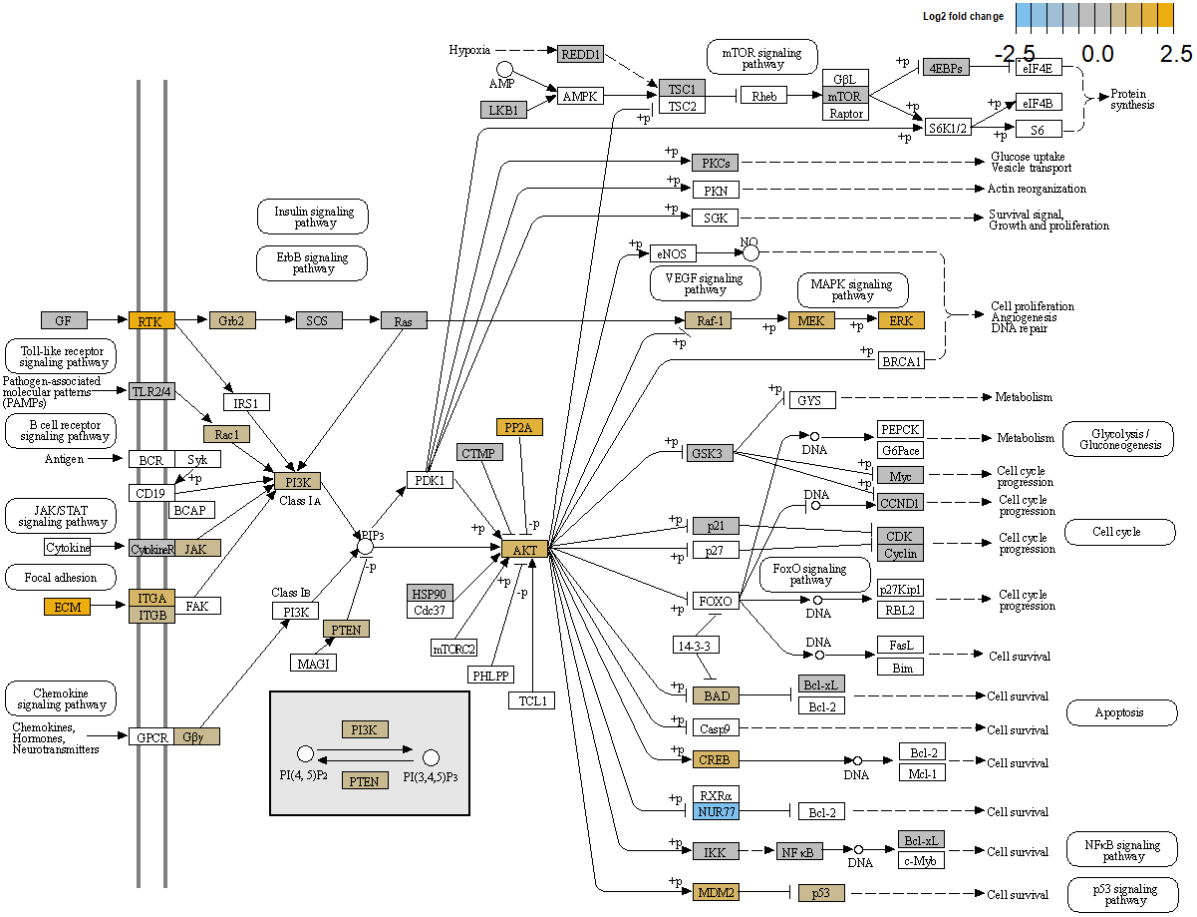
PI3K‐AKT signaling pathway by nSolver Software (PathView). The PI3K‐AKT signaling pathway has pleiotropic effects on both normal and cancerous cellular processes, including but not limited to cell migration, survival, growth, proliferation, and metabolism. PI3K upstream activates AKT by generating PIP3, while PTEN is a negative regulator reverting PIP3 to PIP2. Multiple kinases, such as PDK1 and mTORC2, activate AKT, stimulating cell survival and proliferation and promoting tumor growth. BAD, FOXOs, and MDM2 are just some of the downstream targets of AKT. By contrast, AKT is negatively regulated by several proteins, such as PP2A and PHLPP. The colored proteins are those encoded by significantly differentially expressed genes between the two groups in our study. The different color indicates the different level of expression (log2‐fold change) of the target gene in the high‐clinical‐risk group compared to the low‐clinical‐risk group.

*AKT2* emerged among the upregulated genes in the high‐clinical‐risk group. *AKT2* is an oncogene encoding one of the three isoforms of the serine/threonine kinase AKT (AKT1, AKT2, and AKT3) [23, 24]. AKT is responsible for a variety of both normal and cancerous cellular processes, including cell migration, proliferation, invasion, survival, and metabolism [25, 26]. PI3K upstream activates AKT through its ability to generate PIP3, while PTEN is a negative regulator of the PI3K/AKT pathway based on its lipid phosphatase activity to revert PIP3 to PIP2 [27, 28, 29, 30]. Multiple kinases, such as PDK1 and mTORC2, activate AKT, stimulating cell survival and proliferation and promoting tumor growth [31, 32]. PRAS40, BAD, FOXOs, and MDM2 are downstream targets of AKT [33]. By contrast, AKT signaling is negatively regulated by several proteins, such as protein phosphatase 2 (PP2A) and PH domain and leucine‐rich repeat protein phosphatase‐1 and ‐2 (PHLPP1 and PHLPP2) [34]. AKT and PTEN also play a role in PI3K‐indipendent signaling events. There is growing evidence that AKT isoforms have often distinct roles in promoting oncogenic progression [35]. In PC genetically engineered mouse models, AKT1 is responsible for primary tumor growth, whereas AKT2 promotes the development of distant metastasis and disease aggressiveness [36, 37]. PI3K/AKT signaling is elevated in a high proportion of PC patients due to the genetic alterations and deregulated gene expression of PI3K pathway components occurring in as many as 42% of primary and 100% of metastatic PC samples [38, 39, 40, 41, 42]. The PI3K/AKT pathway activates the genomic and non‐genomic AR and other signaling cascades including, but not limited to, MAPK and WNT signaling (Figure 4) [43]. *AKT1* and *AKT2* activating mutations are rare in PC (0.9%), whereas *AKT1* and *AKT2* upregulation is more common, especially in advanced disease (up to 4.5%, 2%, and 4.7%, respectively) which correlates with the Gleason score and invasive progression [44, 45]. Using nonnegative matrix factorization (NMF), Yuen et al. identified a molecular subtype of PC (NMF2) associated with *FOXO*‐mediated transcription signature and PI3K/AKT signaling. The NMF2 subtype included only primary PC [46]. While the role of PI3K/AKT signaling has been largely investigated in mCRPC, where the AKT inhibitor ipatasertib in combination with abiraterone significantly improved the survival in a phase III trial [47], little or none is known about the prognostic and predictive role of *AKT* upregulation in mHSPC. Our finding of *AKT2* overexpression in the high‐clinical‐risk group of mHSPC could suggest the use of a target therapy also in this setting.

To our knowledge, this is the first study to analyze the gene expression profile of mHSPC with the NanoString nCounter assay. The mutation profile of mHSPC is indeed poorly characterized, since sequencing efforts have focalized on either localized PC or mCRPC; different data seem to indicate that the mutation profile of mHSPC lies between localized PC and mCRPC, suggesting that the enrichment of deleterious alterations over time confers survival advantage to cancer cells inducing treatment resistance [48, 49, 50]. Investigating gene expression from tumor biopsy could help to identify PC patients at higher risk of progressing disease, which could benefit more from an intensified treatment or monitoring. The validation of biomarkers in mHSPC is still an unmet clinical need; gene expression profiling from tumor biopsy could be a useful tool to pursuit of this aim. The costs and the expertise needed for this procedure are well‐known disadvantages that will be probably overcome in the next few years.

Although giving an innovative genetic insight into mHSPC, our study has several drawbacks. The small cohort and the retrospective nature are limits of our study. The potential prognostic role of the identified genetic signature needs to be assessed through a prospective large cohort study. Moreover, the cause of the up‐ or downregulation of the genes differentially expressed will be the subject of further investigation as key to prepare the soil for a potential target therapy in mHSPC.

## 5 Conclusions

Our non‐randomized, single‐center, retrospective trial revealed a different genetic signature between high‐ and low‐clinical‐risk mHSPC, with overexpression especially in apoptosis, PI3K and MAPK pathway‐associated genes in the first group. This difference could help to identify new biomarkers useful for the choice of a tailored treatment for patients affected by mHSPC, as well as to discover new therapeutic targets also in the hormone sensitive phase, such as AKT. Further investigations and prospective larger cohort studies are needed to assess the prognostic meaning of the observed data and the exact role of the deregulated genes in the metastatic progression.

## Author Contributions

**Claudia Piombino:** visualization (supporting), writing – original draft (lead). **Cecilia Nasso:** conceptualization (equal), data curation (equal), formal analysis (equal), investigation (equal), resources (equal). **Stefania Bettelli:** data curation (equal), formal analysis (equal), investigation (equal), methodology (equal), project administration (equal), resources (equal). **Samantha Manfredini:** data curation (equal), formal analysis (equal), investigation (equal), resources (equal), visualization (lead). **Maria Giuseppa Vitale:** conceptualization (equal), resources (equal), writing – review and editing (equal). **Stefania Pipitone:** resources (equal). **Cinzia Baldessari:** resources (equal). **Matteo Costantini:** resources (equal). **Albino Eccher:** supervision (equal). **Ilenia Mastrolia:** supervision (equal). **Virginia Catani:** supervision (equal). **Francesca Bacchelli:** resources (equal). **Stefania Ferretti:** supervision (equal). **Massimo Dominici:** funding acquisition (equal), supervision (equal), writing – review and editing (equal). **Roberto Sabbatini:** conceptualization (equal), methodology (equal), project administration (equal), supervision (equal).

## Ethics Statement

Ethical approval was granted by the institutional Area Vasta Emilia Nord Ethics Committee (Italy)—approval number 927/2020, date of approval November 3, 2020.

## Conflicts of Interest

The authors declare no conflicts of interest.

## Supporting information


Figure S1.

Table S1.

## Data Availability

Data are available upon reasonable request.
