# Supplementary material for: A Novel Molecular Profile of Hormone‐Sensitive Prostate Cancer Defines High Risk Patients
Source: Cancer Med. 2025 Feb 20;14(4):e70472. doi: 10.1002/cam4.70472 (PMC11842281; doi:10.1002/cam4.70472)
Supplement: Supplementary file 1 — Figure S1. [file CAM4-14-e70472-s002.docx]

**Fig. S1**


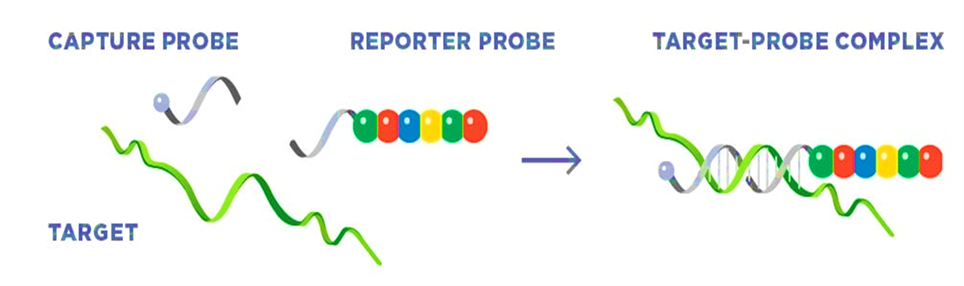


NanoString molecular barcoding technology. Each probe pair consists of a color-coded Reporter and a Capture Probe with target-specific sequences covalently attached. During an overnight hybridization, the specific Reporter and Capture Probes hybridize directly to the single-stranded RNA target molecule in solution to form a double-stranded probe-target complex containing a unique colour sequence for each gene of interest.
