# Supplementary material for: A Novel Molecular Profile of Hormone‐Sensitive Prostate Cancer Defines High Risk Patients
Source: Cancer Med. 2025 Feb 20;14(4):e70472. doi: 10.1002/cam4.70472 (PMC11842281; doi:10.1002/cam4.70472)
Supplement: Supplementary file 2 — Table S1. [file CAM4-14-e70472-s001.docx]

Genes differentially expressed in the high-clinical risk group compared to low-clinical risk group.

| **Gene** | **Log2 fold change** | **Std error (log2)** | **Adjusted p-value** | **Gene set** | **Role in prostate cancer** |
| --- | --- | --- | --- | --- | --- |
| *NR4A1* | -3.05 | 0.40 | 1.29×10^-9^ | MAPK, PI3K | Highly expressed in the LNCaP androgenic hormone-dependent cell line^1^ |
| *FOS* | -3.61 | 1.02 | 9.24×10^-4^ | MAPK | Tumor suppressor^2^ |
| *CTNNB1* | 1.27 | 0.17 | 2.67×10^-9^ | Driver Gene, Wnt | Oncogene^3^ |
| *HIST1H3B* | 3.63 | 0.57 | 8.85×10^-8^ | Driver Gene, Transcriptional Misregulation | Unknown |
| *MAPK1* | 1.51 | 0.25 | 3.30×10^-7^ | MAPK, PI3K, Ras, TGF-beta | PC cells proliferation, migration, and invasion^4^ |
| *SF3B1* | 1.63 | 0.28 | 4.77×10^-7^ | Driver Gene | PC cells proliferation, migration, and viability^5^ |
| *NF1* | 1.33 | 0.24 | 1.20×10^-6^ | Driver Gene, MAPK, Ras | Tumor suppressor^6^ |
| *H3F3C* | 0.78 | 0.14 | 2.13×10^-6^ | Transcriptional Misregulation | Unknown |
| *FN1* | 2.46 | 0.49 | 7.31×10^-6^ | PI3K | PC cells proliferation, migration, and AR expression^7^ |
| *SKP1* | 0.85 | 0.17 | 8.86×10^-6^ | Cell Cycle - Apoptosis, TGF-beta, Wnt | Paclitaxel resistance in PC cell lines^8^ |
| *MDM2* | 1.36 | 0.27 | 9.04×10^-6^ | Cell Cycle - Apoptosis, PI3K, Transcriptional Misregulation | PC progression by p53 degradation^9^ |
| *STMN1* | 2.19 | 0.44 | 1.08×10^-5^ | MAPK | PC cells proliferation, invasion, and metastasis^10^ |
| *PTPN11* | 0.992 | 0.21 | 2.85×10^-5^ | Driver Gene, JAK-STAT, Ras | Tumor suppressor^11^ |
| *PDGFRB* | 1.61 | 0.35 | 3.79×10^-5^ | MAPK, PI3K, Ras | Angiogenesis and PC growth^12^ |
| *WEE1* | 1.89 | 0.42 | 3.89×10^-5^ | Cell Cycle - Apoptosis | Cell cycle arrest, replication fork stabilization, and DNA repair^13^ |
| *WNT5A* | 2.52 | 0.55 | 4.05×10^-5^ | Hedgehog, Wnt | PC cells dormancy in bone^14^ |
| *ASXL1* | 1.29 | 0.29 | 4.37×10^-5^ | Driver Gene | Interaction with AR and AR-dependent transcriptional activation^15^ |
| *HIST1H3H* | 2.24 | 0.50 | 5.90×10^-5^ | Transcriptional Misregulation | Unknown |
| *BNIP3* | 1.75 | 0.40 | 5.93×10^-5^ | Chromatin Modification | Transcriptional repression of apoptosis-inducible factor^16^ |
| *LTBP1* | 2.00 | 0.45 | 6.18×10^-5^ | TGF-beta | Unknown |
| *STAG2* | 1.43 | 0.34 | 1.03×10^-4^ | Cell Cycle - Apoptosis, Driver Gene | Homologous recombination repair^17^ |
| *SFRP2* | 2.25 | 0.54 | 1.22×10^-4^ | Wnt | Induction of an osteoblast-like phenotype in PC cell lines^18^ |
| *DNMT1* | 1.58 | 0.38 | 1.24×10^-4^ | Driver Gene | PC cells proliferation and migration^19^ |
| *RAD21* | 1.01 | 0.24 | 1.32×10^-4^ | Cell Cycle - Apoptosis | Unknown |
| *TBL1XR1* | 0.97 | 0.24 | 1.62×10^-4^ | Wnt | AR co-activator, apoptosis inhibition, tumor growth^20^ |
| *CUL1* | 0.72 | 0.18 | 1.66×10^-4^ | Cell Cycle - Apoptosis, TGF-beta, Wnt | Unknown |
| *ITGA6* | 1.30 | 0.32 | 1.72×10^-4^ | PI3K | PC cells invasiveness and progression^21^ |
| *PRKACA* | 1.02 | 0.25 | 2.13×10^-4^ | Cell Cycle - Apoptosis, Hedgehog, MAPK, Ras, Wnt | Unknown |
| *U2AF1* | 0.95 | 0.24 | 2.40×10^-4^ | Driver Gene | Inhibition of PC cells proliferation by reducing ARV7 mRNA levels^22^ |
| *BAX* | 1.66 | 0.42 | 2.66×10^-4^ | Cell Cycle - Apoptosis | Unknown |
| *H3F3A* | 0.82 | 0.21 | 2.88×10^-4^ | Driver Gene, Transcriptional Misregulation | Unknown |
| *KMT2D* | 1.05 | 0.27 | 3.00×10^-4^ | Driver Gene | PC cell proliferation and migration^23^ |
| *PHF6* | 1.70 | 0.44 | 3.12×10^-4^ | Driver Gene | Unknown |
| *GRB2* | 0.96 | 0.25 | 3.17×10^-4^ | JAK-STAT, MAPK, PI3K, Ras | PC progression; shorter survival if Gleason Score ≥8^24^ |
| *NCOR1* | 1.07 | 0.28 | 3.96×10^-4^ | Driver Gene, Transcriptional Misregulation | Maintenance of mitochondrial membrane potential in PC cells^25^ |
| *AKT2* | 0.56 | 0.15 | 4.23×10^-4^ | Cell Cycle - Apoptosis, JAK-STAT, MAPK, PI3K, Ras | Proliferation of androgen-independent PC cell lines^26^, inhibition of cell migration^27^ |
| *TNFSF10* | 1.19 | 0.32 | 4.53×10^-4^ | Cell Cycle - Apoptosis | Unknown |
| *CDH1* | 1.56 | 0.43 | 6.59×10^-4^ | Driver Gene | Tumor suppressor^28^ |
| *POLB* | 1.44 | 0.40 | 7.71×10^-4^ | DNA Damage - Repair | Base excision repair^29^ |
| *ABL1* | 0.97 | 0.27 | 8.44×10^-4^ | Cell Cycle - Apoptosis, Driver Gene, Ras | PC growth and progression^30^ |
| *NOTCH2* | 0.93 | 0.26 | 8.54×10^-4^ | Driver Gene, Notch | PC growth and development^31^ |
| *SETBP1* | 0.96 | 0.27 | 9.04×10^-4^ | Driver Gene | Unknown |

PC: Prostate cancer

1. Hu YL, Zhong D, Pang F, Ning QY, Zhang YY, Li G, Wu JZ, Mo ZN. HNF1b is involved in prostate cancer risk via modulating androgenic hormone effects and coordination with other genes. *Genet Mol Res GMR* 2013;12:1327–35.

2. Riedel M, Berthelsen MF, Cai H, Haldrup J, Borre M, Paludan SR, Hager H, Vendelbo MH, Wagner EF, Bakiri L, Thomsen MK. In vivo CRISPR inactivation of Fos promotes prostate cancer progression by altering the associated AP-1 subunit Jun. *Oncogene* 2021;40:2437–47.

3. Chesire DR, Isaacs WB. Beta-catenin signaling in prostate cancer: an early perspective. *Endocr Relat Cancer* 2003;10:537–60.

4. Huang B, Zhou D, Huang X, Xu X, Xu Z. Silencing circSLC19A1 Inhibits Prostate Cancer Cell Proliferation, Migration and Invasion Through Regulating miR-326/MAPK1 Axis. *Cancer Manag Res* 2020;12:11883–95.

5. Jiménez-Vacas JM, Herrero-Aguayo V, Gómez-Gómez E, León-González AJ, Sáez-Martínez P, Alors-Pérez E, Fuentes-Fayos AC, Martínez-López A, Sánchez-Sánchez R, González-Serrano T, López-Ruiz DJ, Requena-Tapia MJ, et al. Spliceosome component SF3B1 as novel prognostic biomarker and therapeutic target for prostate cancer. *Transl Res J Lab Clin Med* 2019;212:89–103.

6. Chapman L, Ledet EM, Barata PC, Cotogno P, Manogue C, Moses M, Christensen BR, Steinwald P, Ranasinghe L, Layton JL, Lewis BE, Sartor O. TP53 Gain-of-Function Mutations in Circulating Tumor DNA in Men With Metastatic Castration-Resistant Prostate Cancer. *Clin Genitourin Cancer* 2020;18:148–54.

7. Das DK, Naidoo M, Ilboudo A, Park JY, Ali T, Krampis K, Robinson BD, Osborne JR, Ogunwobi OO. miR-1207-3p regulates the androgen receptor in prostate cancer via FNDC1/fibronectin. *Exp Cell Res* 2016;348:190–200.

8. Yang Y, Lu Y, Wang L, Mizokami A, Keller ET, Zhang J, Fu J. Skp2 is associated with paclitaxel resistance in prostate cancer cells. *Oncol Rep* 2016;36:559–66.

9. Wu M, Cui J, Hou H, Li Y, Liu S, Wan L, Zhang L, Huang W, Sun G, Liu J, Jin P, He S, et al. Novel MDM2 Inhibitor XR-2 Exerts Potent Anti-Tumor Efficacy and Overcomes Enzalutamide Resistance in Prostate Cancer. *Front Pharmacol* 2022;13:871259.

10. Ghosh R, Gu G, Tillman E, Yuan J, Wang Y, Fazli L, Rennie PS, Kasper S. Increased expression and differential phosphorylation of stathmin may promote prostate cancer progression. *The Prostate* 2007;67:1038–52.

11. Tassidis H, Brokken LJS, Jirström K, Bjartell A, Ulmert D, Härkönen P, Wingren AG. Low expression of SHP-2 is associated with less favorable prostate cancer outcomes. *Tumour Biol J Int Soc Oncodevelopmental Biol Med* 2013;34:637–42.

12. Johansson A, Jones J, Pietras K, Kilter S, Skytt A, Rudolfsson SH, Bergh A. A stroma targeted therapy enhances castration effects in a transplantable rat prostate cancer model. *The Prostate* 2007;67:1664–76.

13. Gupta N, Huang T-T, Horibata S, Lee J-M. Cell cycle checkpoints and beyond: Exploiting the ATR/CHK1/WEE1 pathway for the treatment of PARP inhibitor-resistant cancer. *Pharmacol Res* 2022;178:106162.

14. Ren D, Dai Y, Yang Q, Zhang X, Guo W, Ye L, Huang S, Chen X, Lai Y, Du H, Lin C, Peng X, et al. Wnt5a induces and maintains prostate cancer cells dormancy in bone. *J Exp Med* 2019;216:428–49.

15. Katoh M. Functional and cancer genomics of ASXL family members. *Br J Cancer* 2013;109:299–306.

16. Chen X, Gong J, Zeng H, Chen N, Huang R, Huang Y, Nie L, Xu M, Xia J, Zhao F, Meng W, Zhou Q. MicroRNA145 targets BNIP3 and suppresses prostate cancer progression. *Cancer Res* 2010;70:2728–38.

17. Kim MS, Kim SS, Je EM, Yoo NJ, Lee SH. Mutational and expressional analyses of STAG2 gene in solid cancers. *Neoplasma* 2012;59:524–9.

18. Akova Ölken E, Aszodi A, Taipaleenmäki H, Saito H, Schönitzer V, Chaloupka M, Apfelbeck M, Böcker W, Saller MM. SFRP2 Overexpression Induces an Osteoblast-like Phenotype in Prostate Cancer Cells. *Cells* 2022;11:4081.

19. Li Z, Li B, Yu H, Wang P, Wang W, Hou P, Li M, Chu S, Zheng J, Mao L, Bai J. DNMT1-mediated epigenetic silencing of TRAF6 promotes prostate cancer tumorigenesis and metastasis by enhancing EZH2 stability. *Oncogene* 2022;41:3991–4002.

20. Simpson BS, Camacho N, Luxton HJ, Pye H, Finn R, Heavey S, Pitt J, Moore CM, Whitaker HC. Genetic alterations in the 3q26.31-32 locus confer an aggressive prostate cancer phenotype. *Commun Biol* 2020;3:440.

21. Koivusalo S, Schmidt A, Manninen A, Wenta T. Regulation of Kinase Signaling Pathways by α6β4-Integrins and Plectin in Prostate Cancer. *Cancers* 2022;15:149.

22. Cao H, Wang D, Gao R, Chen L, Feng Y. Down regulation of U2AF1 promotes ARV7 splicing and prostate cancer progression. *Biochem Biophys Res Commun* 2021;541:56–62.

23. Lv S, Ji L, Chen B, Liu S, Lei C, Liu X, Qi X, Wang Y, Lai-Han Leung E, Wang H, Zhang L, Yu X, et al. Histone methyltransferase KMT2D sustains prostate carcinogenesis and metastasis via epigenetically activating LIFR and KLF4. *Oncogene* 2018;37:1354–68.

24. Iwata T, Sedukhina AS, Kubota M, Oonuma S, Maeda I, Yoshiike M, Usuba W, Minagawa K, Hames E, Meguro R, Cho S, Chien SHH, et al. A new bioinformatics approach identifies overexpression of GRB2 as a poor prognostic biomarker for prostate cancer. *Sci Rep* 2021;11:5696.

25. Tang L, Zhang L, Liu L, Dong L, Dong Y, Zhu W, Wang H. NCOR1 may be a potential biomarker of a novel molecular subtype of prostate cancer. *FEBS Open Bio* 2020;10:2678–86.

26. Cariaga-Martinez AE, López-Ruiz P, Nombela-Blanco MP, Motiño O, González-Corpas A, Rodriguez-Ubreva J, Lobo MVT, Cortés MA, Colás B. Distinct and specific roles of AKT1 and AKT2 in androgen-sensitive and androgen-independent prostate cancer cells. *Cell Signal* 2013;25:1586–97.

27. Shen Y, Gao Y, Yuan H, Cao J, Jia B, Li M, Peng Y, Du X, Zhang J, Shi J. Prohibitin-2 negatively regulates AKT2 expression to promote prostate cancer cell migration. *Int J Mol Med* 2018;41:1147–55.

28. Imtiaz H, Afroz S, Hossain MA, Bellah SF, Rahman MM, Kadir MS, Sultana R, Mazid MA, Rahman MM. Genetic polymorphisms in CDH1 and Exo1 genes elevate the prostate cancer risk in Bangladeshi population. *Tumor Biol* 2019;41:1010428319830837.

29. Vasquez JL, Lai Y, Annamalai T, Jiang Z, Zhang M, Lei R, Zhang Z, Liu Y, Tse-Dinh Y-C, Agoulnik IU. Inhibition of base excision repair by natamycin suppresses prostate cancer cell proliferation. *Biochimie* 2020;168:241–50.

30. Arora S, Saini S, Fukuhara S, Majid S, Shahryari V, Yamamura S, Chiyomaru T, Deng G, Tanaka Y, Dahiya R. MicroRNA-4723 Inhibits Prostate Cancer Growth through Inactivation of the Abelson Family of Nonreceptor Protein Tyrosine Kinases. *PLoS ONE* 2013;8:e78023.

31. O’Brien R, Marignol L. The Notch-1 receptor in prostate tumorigenesis. *Cancer Treat Rev* 2017;56:36–46.
